# Adaptive Template Reconstruction for Effective Pattern Classification

**DOI:** 10.3390/s23156707

**Published:** 2023-07-26

**Authors:** Su Yang, Sanaul Hoque, Farzin Deravi

**Affiliations:** 1 Department of Computer Science, Faculty of Science & Engineering, Swansea University, Swansea SA1 8EN, UK; 2 School of Engineering, University of Kent, Canterbury CT2 7NT, UK; f.deravi@kent.ac.uk

**Keywords:** instance-based classification, pattern recognition, template reconstruction, image classification, time-series data

## Abstract

A novel instance-based algorithm for pattern classification is presented and evaluated in this paper. This new method is motivated by the challenge of pattern classifications where only limited and/or noisy training data are available. For every classification, the proposed system transforms the query data and the training templates based on their distributions in the feature space. One of the major novelties of the proposed method is the concept of template reconstruction enabling improved performance with limited training data. The technique is compared with similar algorithms and evaluated using both the image and time-series modalities to demonstrate its effectiveness and versatility. Two public image databases, FASHION-MNIST and CIFAR-10, were used to test its effectiveness for the classification of images using small amounts of training samples. An average classification improvement of 2~3% was observed while using a small subset of the training database, compared to the performances achieved by state-of-the-art techniques using the full datasets. To further explore its capability in solving more challenging classification problems such as non-stationary time-series electroencephalography (EEG) signals, a clinical grade 64-electrode EEG database, as well as a low-quality (high-noise level) EEG database, obtained using a low-cost system equipped with a single dry sensor, have also been used to test the algorithm. Adaptive reconstruction of the feature instances has been seen to have substantially improved class separation and matching performance for both still images and time-series signals. In particular, the method is found to be effective for the classification of noisy non-stationary data with limited training data volumes, indicating its potential suitability for a wide range of applications.

## 1. Introduction

With the recent developments in the field of automatic pattern recognition, particularly associated with deep learning techniques, pattern classification performance for a range of modalities (such as image and time-series data) has considerably improved. However, pattern recognition based on limited training data (insufficient to avoid over-training when using deep learning) remains a challenging topic.

In this paper, we propose an instance-based classification method using a novel template reconstruction mechanism, which can achieve good performance for a range of classification problems using a relatively small amount of training data. To demonstrate its generality, the method is evaluated using both image benchmarking datasets as well as more challenging time-series data (EEG recordings).

The limited availability of training data has long been a practical challenge for many pattern recognition problems. This may lead to under-generalization of the feature space and, as such, result in poor classification performance in real-world scenarios. To alleviate this, the algorithm proposed in this work aims to improve pattern recognition without leveraging the addition of training data. This is achieved through a multi-stage adaptive mechanism, incorporating the generation of new templates based on modified versions of existing template data.

Most of the machine learning algorithms depend on similarity-based measurements (explicitly or implicitly) [1]. The within-class and between-class similarities are critical factors for effective classification [2]. High between-class similarity may result in a high false positive rate, while low within-class similarity may produce a high false negative classification rate. To achieve good recognition performance, instance distributions may be examined to isolate and select key instances that could lower between-class similarity and increase within-class similarity. Such subsets of instances can be further remodeled/reconstructed to provide compact representations for each class. This idea is explored and utilized in the proposed technique, by basing the reconstruction process on the mutual influence of query instance and the training templates, which are simultaneously remodeled for every classification attempt.

One advantage of instance-based learning over some other learning methods, such as eager learning algorithms, which perform explicit generalization [3,4] (e.g., neural networks), is their ability to adaptably model the training set by using only a subset of it, hence potentially providing better local approximations.

For example, in a scenario where the size of the training data is changeable (e.g., where additional data enrolments are possible), the templates of interest could be updated by using instance-based recognition methods. However, simply increasing the number of templates without a selection scheme also introduces additional complexities to the system. Therefore, various instance reduction and generation algorithms have been developed to deal with such complexities [5]. The development of these optimization algorithms has had several aims, including (1) increasing the speed, (2) minimizing storage and (3) improving generalization accuracy. Typical modern instance-based learning algorithms where instance selection is employed include the k-Nearest Neighbor (k-NN) and the Support Vector Machine (SVM) algorithms.

Certain time-series data such as electroencephalography (EEG) signals are characterized by their non-stationary behavior [2]; the signal statistics for the same class are often unstable and vary over time. The within-class variance over time, such as longitudinal template ageing effects in person recognition, greatly impacts recognition accuracy. Based on our investigations and other results reported in [6], even a time interval of several minutes between independent recordings could cause considerable template variations. Therefore, data-driven adaptive instance generation can play an important role for concurrently achieving both satisfactory within-class as well as between-class discriminations. To demonstrate the advantages of the proposed method, EEG data provide a challenging test case and two different EEG databases are used to evaluate the effectiveness of the proposed instance-based template reconstruction algorithm.

The main contributions of this paper are summarized below:

(1) A novel instance-based template reconstruction algorithm is proposed for pattern recognition. The algorithm is divided into two phases: Phase I is designed to generate a training set with improved quality for pattern recognition by maximizing the between-class separation. Phase II is designed to further optimize the recognition process. The key innovation of the algorithm is to adaptively modify the training and the probe templates to ensure best use of the available date for establishing the correct class for each matching action. The proposed method can achieve good classification performance by leveraging only a small amount of training data.
(2) The proposed method is found to perform robustly across two popular benchmarking image databases, showing its effectiveness in image classification. For the more challenging classification problem of non-stationary time-series data, the proposed algorithm has also been tested and found to be effective for EEG signal classification, indicating its versatility for wider applications.

The paper is structured as follows: The detailed description of the algorithm is given in Section 2. The effectiveness of the proposed method is evaluated in Section 3, including a number of pattern recognition scenarios for both image and signal modalities, along with a brief comparison with the optimized k-NN and SVM algorithms. In Section 4, a walk-through of the algorithm with a series of illustrations is presented using real EEG data, and an analysis of the computational efficiency of the proposed method is also provided. Section 5 is devoted to overall conclusions, as well as suggestions for future work.

## 2. Instance-Based Template Regeneration

**Figure 1:**
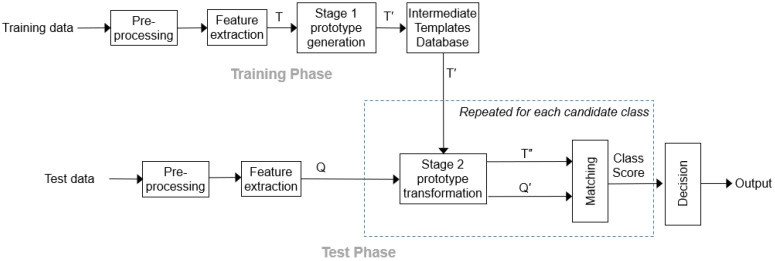
The proposed I-ATR approach.

The Instance-based Adaptive Template Reconstruction (I-ATR) algorithm consists of two phases (cf. Figure 1). Phase 1 (Training Phase) generates intermediate templates by reconstructing the initial templates, so that the between-class separation is enhanced. Phase 2 (Matching Phase) further transforms these intermediate templates based on the query patterns to establish the best class assignment for the given query. Where multiple instances are available for a given query, they can also be transformed. Phase 2 transformations are carried out class-wise so that the distance between the query and the nearest template within that class is the smallest. The following sections describe the proposed algorithm in more detail.

### 2.1. Data Structure

**Figure 2:**
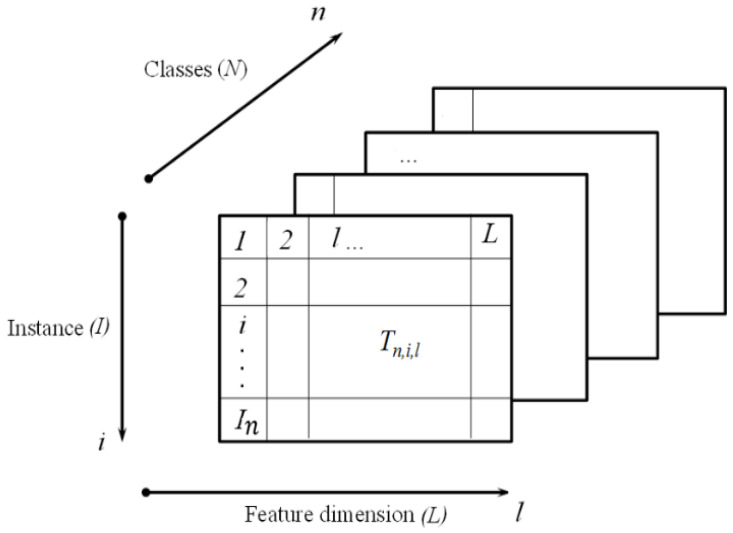
Training set matrix $T_{n,i,l}$ where 1≤n≤N,1≤i≤I(n),1≤l≤L.

Consider an *N*-class classification problem. *T* denotes the instances of the training set, the query (test) instances are denoted as Q, and the instances per class as I(n), where *n* indicates the class label (1 ≤ *n* ≤ *N*). Each instance is an *L*-dimensional feature vector, while each class may have a different number of instances. A three-dimensional matrix $T_{n,i,l}$, could be used to represent the data elements of the feature vector in the training set. Here, i denotes the index of an instance with a specific location, *n* denotes the class to which that instance belongs, and *l* denotes the feature dimension index (1 ≤ *l* ≤ *L*) (c.f. Figure 2). $Q_{r,l}$, (1 ≤ *r* ≤ *R*), represents the query descriptor where *R* is the number of feature instances available per query event. In a typical pattern classification task, *R* = 1; however, for time-series data (such as EEG recordings), multiple feature vectors can easily be generated from the same recording, thus resulting in multiple feature vectors from one recording (*R* > 1).

### 2.2. Phase 1 (Training Phase)

The Training Phase of the I-ATR algorithm is devoted to maximizing the between-class separation of the training set. For a given feature element (*l*) of the training instance (*i*) within each class (*n*), the average distance ${\bar{d}}_{n,i,l}$ between this element and the corresponding feature elements of all the other classes are measured (cf. step 1–step 2 in Box 1). These mean distances ${\bar{d}}_{n,i,l}$ for a given class *m* (1 ≤ *m* ≤ *N*) indicate the distance scores for the corresponding feature elements with respect to the remaining classes as a whole. The elements are then ranked, and a subset of the *K* instances with large between-class distance scores are retained to form the intermediate templates, $T_{n,i,l}^{\prime },$ as shown by step 3–step 4 in Box 1. *K* should be empirically fixed depending on its application.

Box 1 **Training Phase.**

**Training Phase: Maximize between-class Separation**

For *n* = [1, N]

 For *i* = [1, I(*n*)]

   For *l* = [1, *L*]

   **1)** Compute $d_{n,i,l}\left(m,j\right)=distance(T_{n,i,l},T_{m,j,l}$),

   where 1 ≤ *m* ≤ *N*, *m ≠ n* and *1 ≤ j ≤ I(m).*

   // any appropriate distance metric may be used here. In

   // this work, *distance (x, y) = |x-y|* is used.

   **2)** Compute the mean of the resulting distances:

   ${\bar{d}}_{n,i,l}\left(m\right)=\frac{1}{I\left(m\right)}\sum_{j}d_{n,i,l}\left(m,j\right).$

End of for-loops.

For *n* = [1, *N*]

   For *l* = [1, *L*]

   **3)** $index={arg\;sort}_{i}({\bar{d}}_{n,i,l},descending)$.

   **4)** $T_{n,i,l}^{\prime }=T_{n,index\left(1:K\right),l}$, where ***K*** is the number of

   intermediate templates to be generated for each class.

End of for-loops.

// The resulting feature set $T_{N,I,L}^{\prime },\;\mathrm{where}\;I=index\left(1:K\right),K<I(n)$ now forms intermediate templates with relatively large between-class distances, which will be used in Phase 2 of the process.

It may be noted that the Training Phase has some similarities with the computation of Hausdorff Distance [7]. In the proposed method, the absolute distances between elements of the feature vectors from different classes are measured, instead of the L_2_ distance between the feature vectors. This mechanism has been found to generate instances with much better between-class separations. As the feature elements originate from different feature vectors, these intermediate templates may not have exact equivalents in the original set of feature vectors. However, these reconstructed vectors will always be on the surfaces of or within the bounding hyper-plane of the original training data set.

### 2.3. Phase 2 (Matching Phase)

The Matching Phase of the proposed method further transforms the intermediate templates $T_{n,i,l}^{\prime }$, producing $T_{n,i,l}^{\prime \prime }$ to minimize their distances from the query instances. Similar to the Training Phase, element-wise distances $d_{n,i,l}$ between the intermediate template $T_{n,i,l}^{\prime }$ for each candidate class and the query patterns $Q_{r,l}$ are measured (cf. Step 5, Box 2). However, in the Matching Phase, only the feature elements contributing the smallest distances are used to form the transformed feature vectors $T_{n,l}^{\prime \prime }$. The process is illustrated by Step 6–Step 9 in Box 2. Where multiple instances from the same query observation are available (such as in EEG recordings), the transformation is also applied to the query instances producing transformed query vectors $Q_{r,l}^{\prime }$. The distance between $T_{n,l}^{\prime \prime }$ and $Q_{r,l}^{\prime }$ represents the score for the class under consideration. The process is repeated independently for each class, thus ensuring the best possible match between the training and the test data for each class. The class achieving the lowest distance score (s) (hence, the most similar) is assigned to the query pattern, as is shown by Step 10. The full algorithmic process for the Matching Phase is illustrated in Box 2. This phase has been found particularly effective in alleviating the longitudinal template variations for the time-series EEG data classification.

Box 2 **Matching Phase.**

**Matching Phase: Preferential matching between the query sample and the stored templates**

 // Let $T_{n,j,l}^{\prime }$ are the intermediate templates (from the Training Phase) and $Q_{r,l}$ be the query feature vectors from one observed sample $T_{n,i,l}^{\prime }$ has *K* samples per class generated in the Training Phase and $Q_{r,l}$ has *R* instances extracted from an observation

 For *n* = [1, *N*]

   For *i* = [1, *K*]

    For *l* = [1, *L*]

      **5)** Compute $d_{n,i,l}\left(r\right)=distance(T_{n,i,l}^{\prime },Q_{r,l})$,

       where 1 ≤ *r* ≤ *R*.

End of for-loops.

For *n* = [1, *N*]

 For *l* = [1, *L*],

   **6)** $index={arg\;sort}_{i}(d_{n,i,l},ascending)$.

   **7)** $T_{n,l}^{\prime \prime }=T_{n,index\left(1:F\right),l}$, where *F* is the number of finally

    preserved instances for the training set per class.

 

   // Define the instance index for query set as index_q:

   **8)** $index\_q={arg\;sort}_{r}(d_{n,i,l}(r),ascending)$.

   **9)** $Q_{s,l}^{\prime }\left(n\right)=Q_{index_{q\left(1:S\right)},l}$, where *S* is number of modified

    query vectors to be reconstructed.

End of for-loops

 // Classification Stage

 For *s* = [1, *SN*]

   **10)** Compute $\tilde{n}\left(s\right)=\arg{min}_{s}\;distance(T_{n,l}^{\prime \prime },{\;Q}_{s,l}^{\prime })$

   // $\tilde{n}\left(s\right)$ is the class closest to the query under consideration

 End of for-loop

 $overall\;decision=majorityVote(\tilde{n}(s))$.

## 3. Case Study Evaluations

In this section, the proposed I-ATR method is evaluated using both image and time-series modalities to explore its robustness across different application scenarios. Two major subsections are subsequently presented, where each subsection is devoted to evaluating the I-ATR classification method using two databases.

### 3.1. Image Data Classifications

Two popular benchmarking databases, namely FASHION-MNIST [8] and CIFAR-10 [9] datasets, are used to evaluate the I-ATR algorithm. Its classification performance, as well as comparisons with other state-of-the-art systems for the related databases, are reported in the following sections. We selected these two public datasets for two main reasons: (1) they are popular benchmarking datasets that have been extensively used in the related fields, convenient for comparative analysis; and (2) these two datasets are particularly helpful in reflecting the advantages of the proposed method.

#### 3.1.1. Evaluation Using Greyscale Images

We employed the FASHION-MNIST dataset to explore the effectiveness of the proposed method for greyscale image classification. FASHION-MNIST is an image dataset consisting of a training set of 60,000 samples and a test set of 10,000 samples. Each sample is a 28 × 28 grayscale image, associated with a label from 10 classes. Compared with the MNIST database, the FASHION-MNIST is often considered as a more challenging database for classification problems [8].

In order to adapt and integrate the proposed recognition algorithm with the image modality, the 28 × 28 grayscale image is reshaped into 784 element vectors. A training set of 6000 × 784 is therefore created for each class, and the test samples form a 1000 × 784 matrix per class. Since only one sample is available per query, image augmentation is applied to generate additional images. In this case, we simply applied random rotation (±5°) to the given query image to increase the query set. It has been found that only one augmented image is adequate.

The main principle of the proposed classification algorithm is to enhance the informative content of the data by optimizing the inter- and intra-class separations through reconstructing the samples.

**Figure 3:**
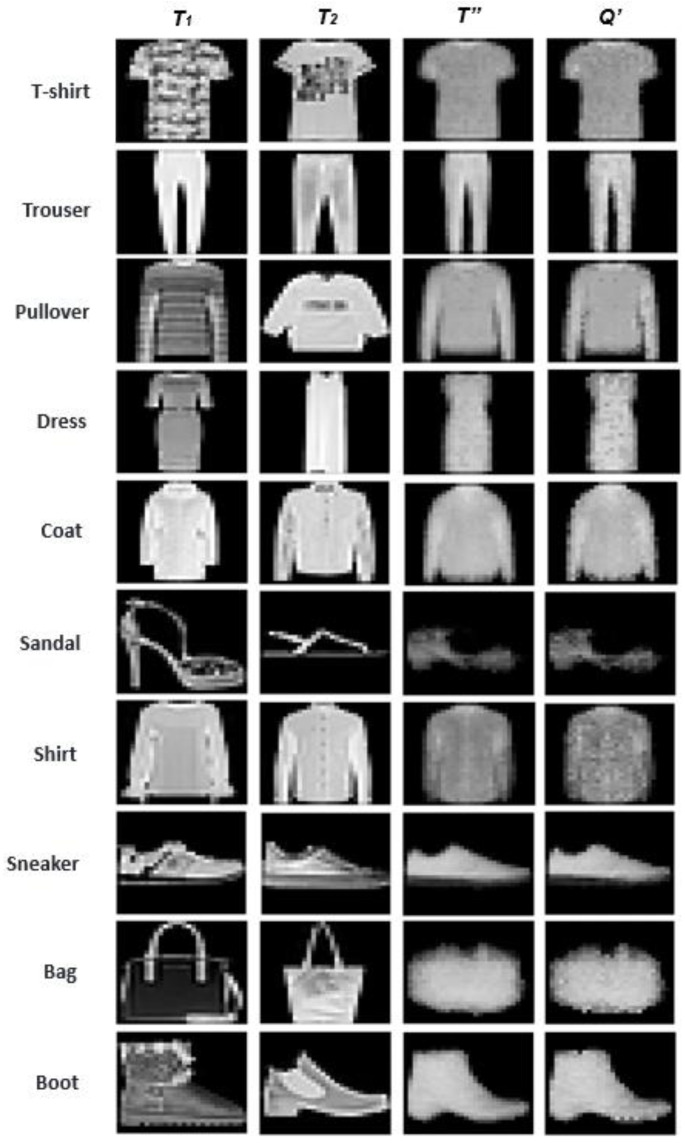
An illustrative display of the images before and after performing the I-ATR transformation. Two random training samples (*T*) from each class are included for visual comparisons. The non-contributing patterns of the images were automatically ignored in the reconstructed *T”* and *Q’* samples.

Figure 3 provides a visual illustration of the images before and after template reconstruction. The first two columns display two randomly selected initial samples from each class (*T*_1_ and *T*_2_, cf. Box 1); the remaining two columns show the reconstructed training and test samples (*T″* and *Q′*, cf. Box 2). As can be observed from the samples, after the transformation, the resulting training and query templates tend to ignore the irrelevant details, while preserving and enhancing the effective boundaries. Both the reconstructed training and query samples tend to converge to similar instances (images).

**Figure 4:**
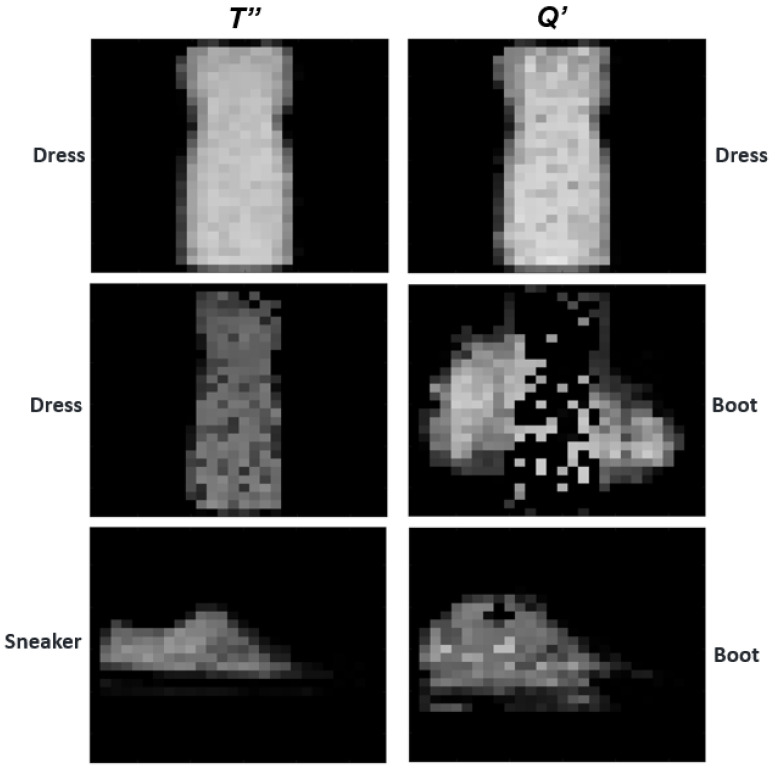
Comparison of reconstructed templates and queries. When the test attempts are from different classes, the resulting image is not recognizable and is hence rejected by the I-ATR classifier.

We also conducted a series of experiments to explore its effectiveness while the system was present with an image from a different class. A few visual comparisons using images from different categories are illustrated in Figure 4. In particular, the Boot category is purposely used as the query against some different training categories for this illustration, as it has been noticed that the Boot is the class that usually caused most of the erroneous classifications; it also tends to be confused with the Sneaker category. In Figure 4, we also highlight the impact toward the reconstructed training templates while images from the same class and a different class are presented separately for testing. For example, while feeding the Boot sample query to the Sneaker training category, the resulting reconstructed training template is much less like the genuine case (cf. Figure 4 Sneaker *T″* and Boot *Q′* for this comparison). It has been found that the proposed method was able to maintain low false positive rates (<1%) for the FASHION-MNIST dataset by reconstructing the training templates.

The overall performance of the proposed method for distinguishing the 10 classes from the FASHION-MNIST dataset is explored. An average classification accuracy of around 99% was achieved using the proposed recognition algorithm—by far the best reported performance for this database. It is worth noting that this accuracy was achieved by training with only seven randomly selected images for each category from the training set and tested by one random image. This experiment was repeated several times to obtain the mean accuracy.

#### 3.1.2. Evaluation Using Color Image Data

The CIFAR-10 dataset [9] has been used to study the effectiveness of the proposed method for color image classification. The CIFAR-10 dataset contains 60,000 32 × 32 colour images in 10 classes, with 6000 images per class. There are 50,000 training images and 10,000 test images. The database is well-balanced, each class in the training set consists of exactly 5000 images, and the remaining 1000 images can be used for testing.

**Figure 5:**
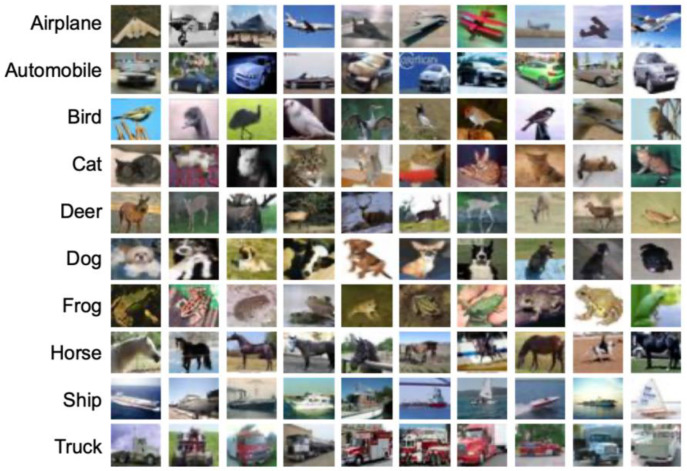
A few random samples from the CIFAR-10 dataset.

Figure 5 presents 10 random images from each class of this dataset. The classes are mutually exclusive. There is no overlap between automobiles and trucks. “Automobile” includes sedans, SUVs, etc., and “Truck” includes only big trucks. Neither includes pickup trucks [9].

Following a similar pre-processing approach for the FASHION-MNIST dataset, we directly employed the three channel RGB images from the dataset for classification without conducting any further feature extraction; each 32 × 32 color image has been reshaped into an image vector with the length of 3072 dimensions (pixels). In this way, each observation is one image, together forming a 5000 × 3072 matrix for each class. The test set follows the same reshaping approach.

As it can be observed from Figure 5, compared to the FASHION-MNIST grayscale dataset, the images in CIFAR-10 dataset have arguably less distinguished edges. Hence, this database with RGB images may be potentially more challenging than FASHION-MNIST for classification.

**Figure 6:**
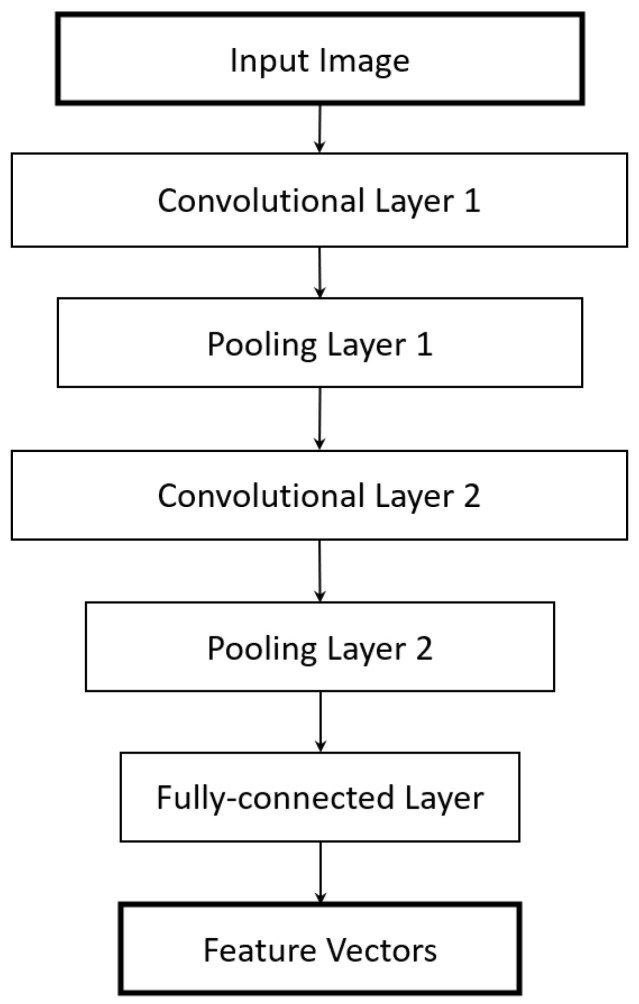
The overall architecture of the employed CNN for the NN-based feature extraction. The final fully connected layer produces a vector of size 10.

When using the raw pixel vectors as the training template, as was the case for the grayscale MNIST-FASHION data, we obtained an average accuracy of about 90% for the color dataset. Instead, for the CIFAR-10 dataset, we also used a 2D convolutional neural network (CNN) to generate the input training vectors for the I-ATR scheme. The architecture of this CNN network is illustrated by Figure 6. The 3-channel RGB image is first transformed into a 16-channel feature map via the first convolutional layer. The height and width of the output are then halved by 2D Max pooling. As more layers are added, the size of the feature map becomes smaller. The fully connected layer at the end generates a vector of size 10 for each image, which are used as the input for the template reconstruction method proposed in this work.

Based on the confusion matrix analysis for the CIFAR-10 dataset images, it is observed that most of the erroneous predictions were between class 3 (bird) and class 9 (ship). One possible explanation for the wrong predictions could be that some images of birds and ships share similarities in terms of the narrowness and peaky shape in their frames (cf. Figure 5).

Using the I-ATR algorithm, even higher classification performance can be achieved with three samples for testing (one original, two augmented) where the performance managed to reach more than 98% for the mean classification rate. It is worth emphasizing that for each recognition, only small proportions of the data (ten images) were utilized to reconstruct the training samples and achieved comparable performance with the state-of-the-art reports. The results demonstrate that the proposed method is not only effective in image classification with feature transforming, but also drastically reduced the required the amount of data needed for training. This could be a significant advantage over the current deep learning methods in some image recognition scenarios.

#### 3.1.3. Comparison with the State-of-the-Art Results

The image classification performances using these two public benchmarking databases have also been compared with other state-of-the-art results reported in the literature [10,11]. Table 1 lists some recent classification accuracies reported, along with the best average performances achieved in this study (far-right column). For example, in [12], a fine-tune Differential Architecture Search (DARTS) method was proposed to balance the trade-off between the number of parameters and classification accuracy. In [13], a Sharpness-Aware Minimization (SAM) method was proposed to improve the efficiency in a min–max optimization problem during the gradient descent computations. This is based on the assumption that the parameters lie in neighborhoods having uniformly low loss. The Mixed Sample Data Augmentation (MSDA) in [14], which combines multiple augmentation methods with complementary properties, and the Fractional Max-Pooling method in [15], replaces the convolutional layers with a fractional version of max-pooling to reduce overfitting. The exploration of neural net architectures also received much attention, e.g., in [16], which argues that by simply replacing the max-pooling with a convolutional layer, improvements can be achieved in object detection tasks. Network weight initialization has been explored in [17], where a layer-sequential unit-variance (LSUV) initialization was proposed. It is worth pointing out that all these state-of-the-art results were obtained using deep learning techniques, which often take a few hours for the model training. The proposed method, on the other hand, only needed a few seconds for training and achieved better performance.

### 3.2. Classification of 1D Time-Series Data

In this section, three evaluation studies are presented to assess the effectiveness of the I-ATR method for the classification of 1D time-series data. Unlike the 2D images, the EEG signal in particular, due to its non-stationary nature, could be more challenging for successful pattern recognition. In this section, two EEG databases, one publicly available, the other self-collected, are used to further evaluate the proposed method. The impact of template ageing is investigated here.

Section 3.2.1 reports the evaluations based on a publicly available benchmarking database, namely the EEG Motor Movement/Imagery Dataset (EEG MM/I), collected using the BCI2000 instrumentation system [18]. The results for person recognition of 105 selected classes (subjects) [19] from the MM/I database are presented.

In Section 3.2.2, a locally collected dataset, namely the Mobile Sensor Database (MSD), particularly developed for this exploration, has been used. A single low-cost dry sensor system was deployed, making the data in the MSD more challenging. For the MSD dataset, the signal quality is low and only a relatively small amount of training data are available. A well-established feature, based on wavelet transform, was employed for the analyses presented in this study [20].

#### 3.2.1. Evaluation Using MM/I Dataset for Person Recognition

This section explores the effectiveness of the I-ATR algorithm in person recognition problems and compares its performance with the classical 1-NN and SVM algorithms. To ensure at least 2 min of EEG recording is available for each subject, 105 subjects (of 109) were selected from the MM/I dataset. The experimental studies reported here used wavelet-based features as described in [20].

In the work reported here, only the EEG data from the Oz electrode were used to simulate application scenarios where ease of deployment and fast data processing are important. The experimental arrangement is briefly described below:

(1) EEG signals were segmented into multiple 4 s overlapping windows (50% overlapping).
(2) The wavelet packet decomposition (WPD) [21] was carried out for each time-domain window up to level 3. The resulting level 3 wavelet coefficients between 0 and 60 Hz were extracted (each approximately corresponds to a bandwidth of 10 Hz).
(3) The variance in the wavelet coefficients in each window was used as the feature.
(4) The I-ATR algorithm was then invoked for the template generation and classification process.

##### Parameter Optimization

Prior to the invocation of the I-ATR algorithm, the EEG recordings were segmented into 240 windows (I=240). Note, the training set contained two two-minute recordings for each run. The generated data (reconstructed feature instances) for training when K=160 were found to produce the highest recognition rate.

For person recognition using the MM/I dataset, the optimal proportion of the retained reconstructed instances is empirically established. Recognition accuracies were calculated by the leave-one-out three-fold cross-validation approach using in total 6 min of EEG recording (three runs) from each task. The volume of the retained newly constructed feature vectors is defined as *P%* (of the available total), which corresponds to the parameter *K* in Step 4 of the Training Phase shown in Box 1.

**Figure 7:**
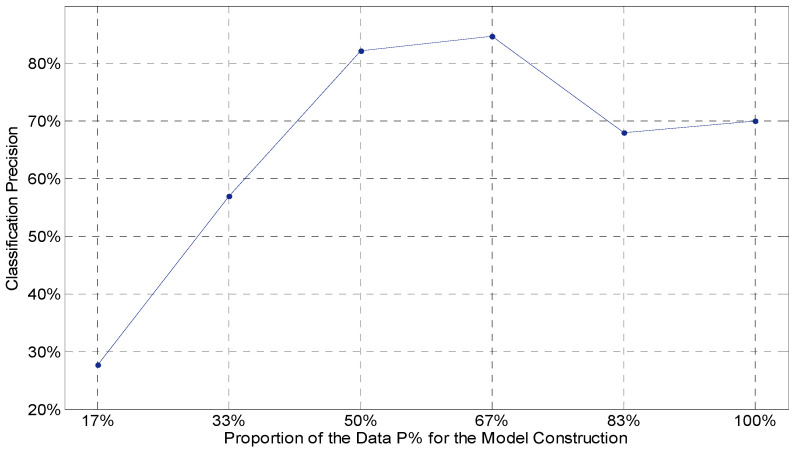
Preliminary tests for optimizing *P*%. The analysis was carried out by dividing the training set into six roughly equal size portions and the preserved data for training were incrementally tested.

Figure 7 shows the change in classification accuracy, while *P*% of the initial number of instances was generated as the intermediate templates for the Training Phase of the I-ATR algorithm. As the preliminary exploration for parameter optimization, only a small subset of the dataset (Subject_1–Subject_10) was used to investigate the overall impact of the retained volume of the reconstructed feature instances for the Matching Phase processing.

The results indicate that higher performances are achieved when the retained volume of the reconstructed intermediate templates is about half to two thirds of the available training data. Therefore, as a rule of thumb, roughly 2/3 of the reconstructed templates will be retained for the next stage of the algorithm. This parameter, however, should be dependent on the quality of the database and the application scenario; for low-quality and noisy signals, such as the Mobile Sensor Database, a different value of *P* may be optimal.

##### Person Identification for MM/I

Following the empirical optimization of parameter *P*, only 66.67% of the training subset of the MM/I dataset samples in their reconstructed form are retained for subsequent evaluation of the proposed method in the Matching Phase. Comparisons are made with the classical k-NN (k = 1) and non-linear SVM [22], as both of them are popular instance-based classification algorithms. The parameters of the k-NN and SVM, i.e., k = 1 for k-NN and second-order polynomial kernel for SVM, are found to be the best performing parameters in this experiment set. We did not conduct performance comparison with deep neural nets in this case study for three major reasons: (1) the available data per class for the MM/I dataset are not large enough for the training to converge, (2) the Mobile Sensor Database used for comparison in performance is smaller and more challenging; 3) the proposed algorithm is particularly effective in handling the classification problems when only a small amount of data were available.

**Figure 8:**
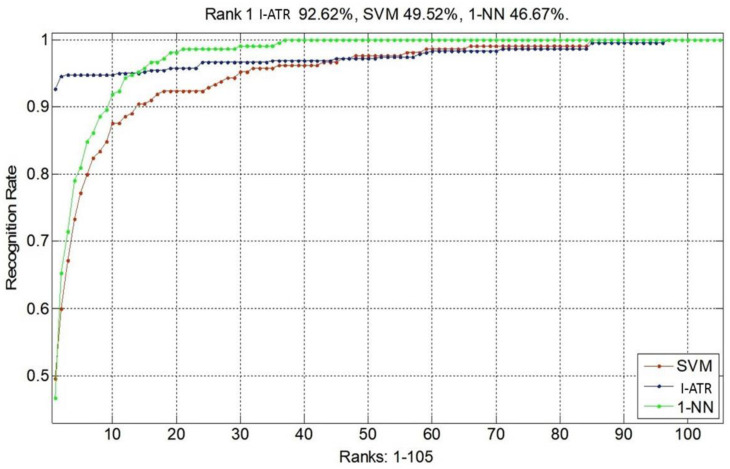
Average recognition performances with three algorithms, using the MM/I database with 105 subjects. The SVM with second-order polynomial kernel and 1-NN are optimized for this scenario.

Figure 8 presents the resulting cumulative match characteristic (CMC) [23] curves for person recognition using the EEG MM/I dataset. The I-ATR algorithm resulted in the best identification rate of 92.6%. The 1-NN classifier produced higher recognition rates only after the top 13 accuracies. Both the SVM and 1-NN had less than 50% top 1 accuracies.

##### Person Verification for MM/I

**Figure 9:**
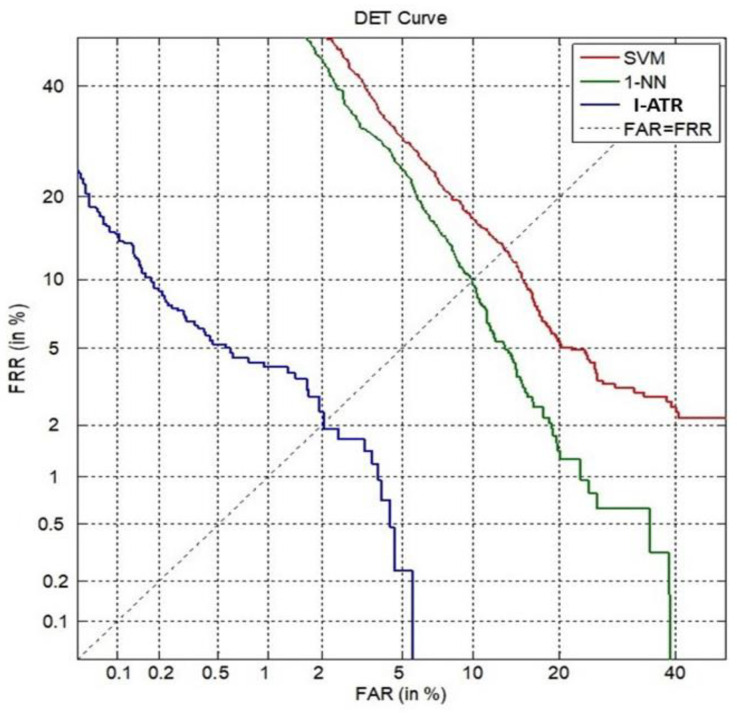
DET curves of three learning algorithms, using the MM/I database with 105 subjects.

Figure 9 presents the performance comparison of the three algorithms using the detection error trade-off (DET) characteristics [24]. From the perspective of biometric person verification, the Training Phase and Matching Phase of the I-ATR algorithm can be viewed as aiming to reduce the false acceptance rate (in Training Phase), i.e., reducing the between-class overlaps in the training set, and the false rejection rate (Matching Phase), i.e., enhancing the provisional within-class matching between the training classes and query, respectively.

For the I-ATR algorithm, each feature vector was generated from 4 s recordings of the EEG signal producing 640 samples, at 160 Hz. However, for the SVM and 1-NN algorithms, 6400 samples per window were used to achieve their best performances. Figure 9 shows an approximately 2% averaged equal error rate (EER) for the I-ATR algorithm, whereas for the 1-NN and SVM classifiers, the averaged EER values were approximately 10% and 14%, respectively.

#### 3.2.2. Performance Using Mobile Sensor Database

The person recognition performance of the I-ATR algorithm was tested using the locally collected Mobile Sensor Database to show the impact of a more challenging scenario. This database was primarily collected to explore the EEG template ageing effect in biometric scenarios. The data collection was aimed to model a real-life application, using a low-cost single dry sensor with a relatively long time interval (three to eight weeks) between the sessions. The EEG data recorded from 30 participants (classes) were used for this study. For the multi-session evaluation, 4 s of continuous data segments were randomly selected from one session (1 min recording) to form a set of the query attempts, and all the data from the other 1 min session were used for training the system. The key features of this database are as follows:

(1) Data were captured using a gaming-grade single dry electrode, positioned at Fp1, designed for ease of deployment (NeuroSky MindWave [25]).
(2) Data were collected from 30 individuals (age ranges from 21 to 55).
(3) Participants were required to engage in a simple sub-vocal number-counting activity (with eyes closed), i.e., the subject sat in a silent room counting numbers, while EEG data were recorded.
(4) Data were collected in two sessions, with the time interval between the sessions ranging from three to eight weeks.

##### Longitudinal Template Ageing Effect

The design of the I-ATR algorithm was in part motivated by the challenge of the template ageing effect in biometric recognition, especially where limited data are available for training [6]. In this section, the impacts of using EEG data from different sessions with respect to different classification algorithms are presented.

Table 2 shows the impact of template ageing when using EEG recordings from the Mobile Sensor Database for person identification. The same parameters as in the last section were used for the best performing k-NN and SVM classifiers. For the single-session scenario, the 60 s recording was split into five non-overlapping segments to enable cross-validation. The average accuracies when using SVM with second-order polynomial kernel and 1-NN classifiers have been found to be comparable at around 93%.

In the case of multi-session data analysis, the entire Session 1 data (60 s) were used for training. A randomly selected segment of the data from Session 2 was then used for testing. The training–testing order was then swapped, and the results averaged. The impact of template ageing is found to be significant, as is shown by the single session vs. multiple session accuracies as reported in Table 2. Only around 10% recognition accuracy was achieved with the conventional classifiers.

The separate impact of the two phases of the I-ATR algorithm is also noticeable. As it is illustrated in Table 2, with only the Training Phase implemented, the template ageing effect has been alleviated substantially for the multiple session case compared to 1-NN and SVM. This suggests the great effectiveness of constructing new feature vectors with better between-class separation. By only applying the Matching Phase, similar considerable improvement was also observed; however, due to the absence of the Training Phase to reduce the potential between-class similarity, the false acceptances were not effectively limited, as the feature instances between the classes in the training set were not necessarily well separated. It is clear from the results that when both the phases are employed, the performance improves significantly.

##### Identification Scenario Using MSD

**Figure 10:**
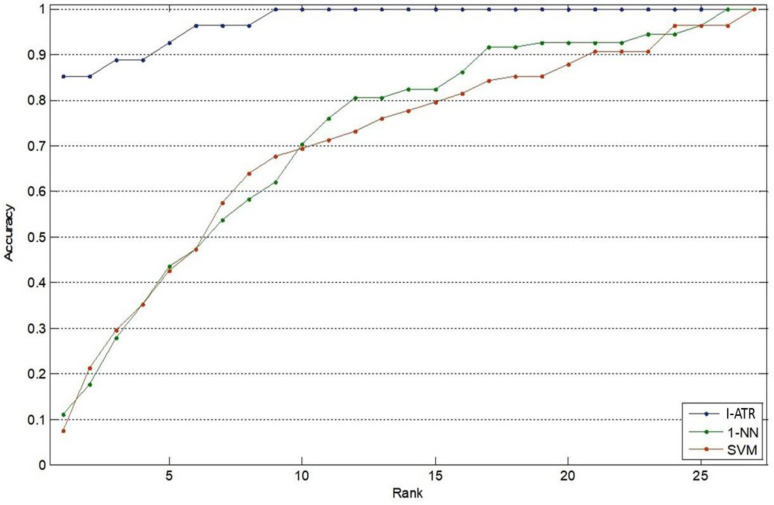
CMC curves of the three learning algorithms, using the Mobile Sensor database, captured from 30 subjects using a single EEG sensor.

The CMC results of the I-ATR algorithm are shown in Figure 10 for the identification scenario. The recognition results obtained using the conventional classifiers are also included for comparison.

The results for these plots were generated using the Session 1 EEG data (60 s recording) for classifier modelling and 4 s recordings from Session 2 as query sets. The parameter I, i.e., the total number of feature instances available, is set to 240. The retained number of instances in the Matching Phase is F=4 for both the training and query sets. As may be observed in Figure 10, the I-ATR method provided much higher accuracy (e.g., 85.71% for rank-1) than the conventional classifiers.

##### Verification Scenario Using MSD

**Figure 11:**
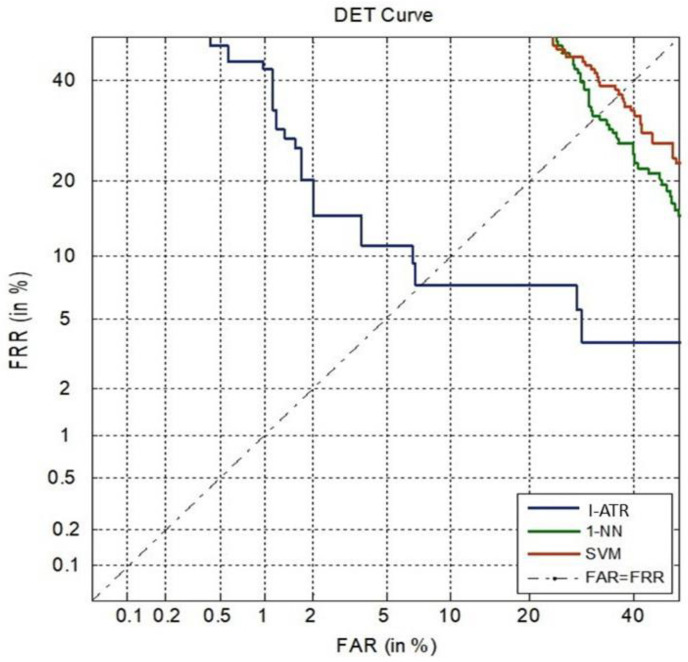
DET curves of three learning algorithms, using the MSD with 30 subjects.

The DET curves for the Mobile Sensor Dataset are presented in Figure 11. Due to the challenging experimental setup (limited and noisy EEG data from a low-cost single dry sensor, cross-session test data), the EER values of all the illustrated algorithms were found to be not as low as those with the MM/I dataset (cf. Figure 9). However, it is clear that the I-ATR still produced much superior EER compared to the other two conventional classifiers. The EER of I-ATR method is around 7.5%, whereas both the 1-NN and SVM schemes’ EER values are higher than 30%.

The results in Figure 9 and Figure 11 highlight the difference in the data quality between the MM/I dataset and the Mobile Sensor Database. The EER using I-ATR for the MM/I database was as low as 2%, whereas for the Mobile Sensor Database with only 30 subjects, it was about 7.5%.

## 4. Discussion

In this section, we provide some discussions of the I-ATR approach, to illustratively demonstrate the method in a step-by-step manner.

### 4.1. Visualization of Feature-Space Transformation

**Figure 12:**
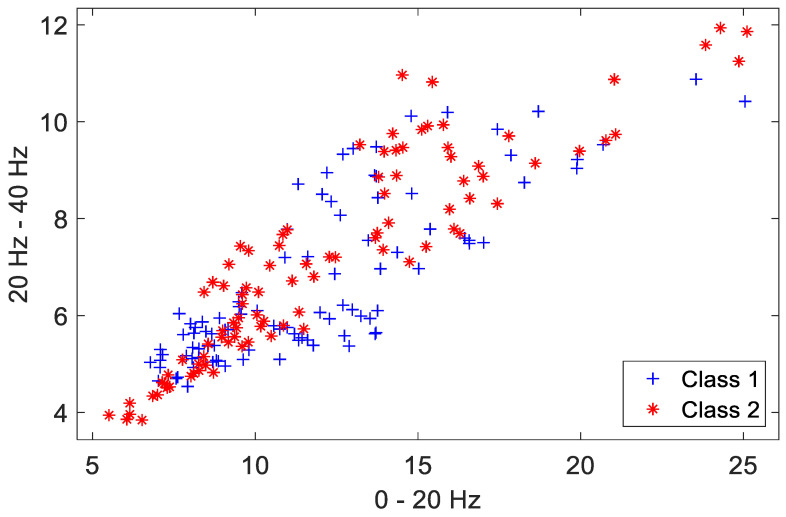
Original template patterns. Class 1 and Class 2 indicate features of the two classes.

A two-class person recognition problem is constructed using data from two randomly selected subjects (hereinafter referred to as Class 1, Class 2) of the MSD for this experimental analysis. The initial pattern distribution of the first two dimensions in the feature space (12 dimensions in total for each feature vector, L=12) for the two classes is shown in Figure 12. The distribution of the extracted features from the instances in the training set is depicted, with each class containing 95 feature instances. The two dimensions shown in the figure along the axes correspond to their approximate frequency ranges in the wavelet domain. Clearly, these initial features are quite intertwined in the pattern space.

Improving the between-class discrimination of the training set is achieved in the Training Phase of I-ATR, by selectively reducing the number of instances and reconstructing new feature vectors for each class. Note that reducing the number of reconstructed features serves as a supplementary step to better separate the overall distribution between classes, only if the initial features were found too intertwined in the feature space. This supplementary step has been found particularly effective to reduce the false acceptance rate for challenging imposter queries.

**Figure 13:**
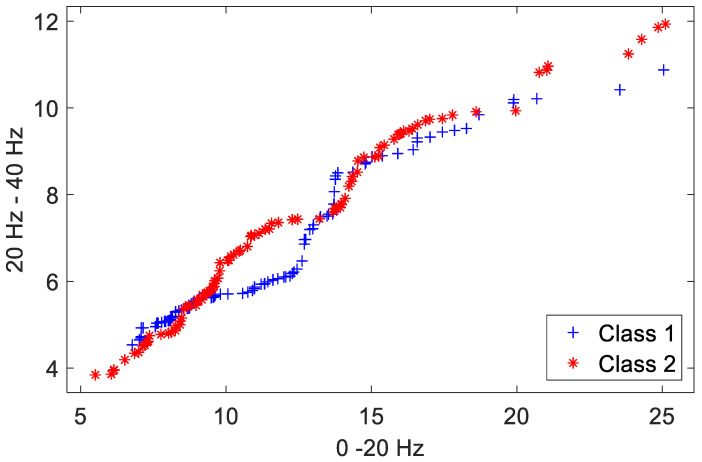
New training set patterns after Training Phase. Class 1 and Class 2 indicate features of the two classes.

Figure 13 shows the resulting patterns after the completion of the Training Phase. The between-class distances of feature elements were ranked, and new feature vectors reconstructed. It is worth emphasizing that the between-class distance measurements were operated and monotonically sorted within each dimension. Compared to the patterns in Figure 12, it is clear that the between-class separation for most of the reconstructed patterns in the training set is much improved after the Training Phase.

The Matching Phase of the I-ATR algorithm aims to minimize the distance between the given query and the transformed training features of the corresponding class, by further transforming the vectors from both ends (training and query samples). This process was repeated for each of the candidate classes. The algorithm thus selected the best templates from the training set $T_{n,j,l}^{\prime }$ and constructed a further reduced and reconstructed subset $T_{n,index\left(1:F\right),l},$ with *F* being the number of retained instances for a given training class. Transformation is also applied to the query set when multiple instances are presented and a subset of the query set will then be constructed for each class in the training set (the number of preserved templates denoted as *S*, *S* = *F* in this study).

**Figure 14:**
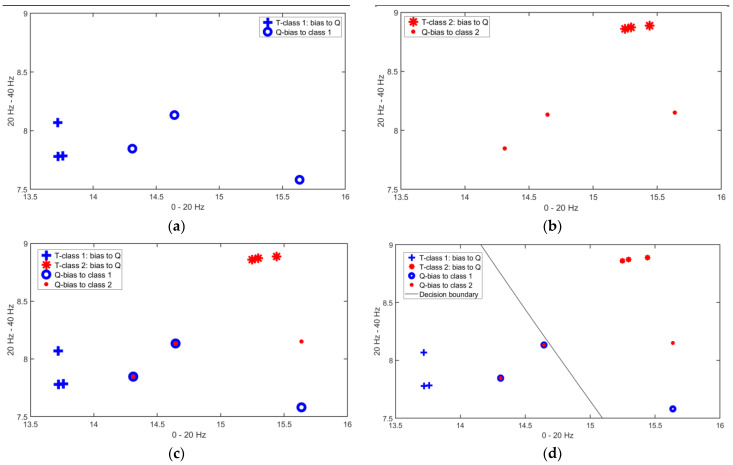
Illustration of the Phase 2 process of the I-ATR algorithm using only the first 2-dimensions of the feature vector for a 2-class problem, where the query Q is from Class 1. (**a**) T and Q are biased for Class 1. (**b**) T and Q are biased for Class 2. (**c**) The training-query features for the two-class scenario merged together. (**d**) The classification outcome.

Figure 14 shows the Matching Phase process for this two-class recognition problem. In this figure, the genuine class (ground truth) of the query belongs to Class 1. The best three reconstructed templates (features) were selected for both the training and the query sets, i.e., the final sets are $T_{n,f,l}^{\prime \prime }$ and $Q_{s,l}^{\prime }$, where N=2, F=S=3 and L=2 for this classification. The first block in Figure 14 shows the resulting features after the Matching Phase for the training set of Class 1 (blue crosses) and the corresponding query feature points (blue circles). The best three feature points were kept after within-class distance ranking. The second block (Figure 14b) illustrates the adaptably reconstructed training feature set and its corresponding query set for Class 2. The feature set of Class 2 and the constructed query set for Class 2 are represented by red symbols.

The final step of the Matching Phase is to measure the absolute distance between the reconstructed query set and the training set for each class; the decision-making is then based on the nearest feature points. As is shown in the last block of Figure 13, for this two-class classification problem, it has been found that out of six query feature points (three points for each class), four of them are classified as belonging to Class 1 (67% for Class 1 vs. 33% for Class 2), which made the decision favoring Class 1 (the same as the genuine class of the query).

Compared to conventional classification approaches, the major difference of the proposed method lies in the element-wise template reconstruction for improved class separation, followed by the decision-making based on reconstructed feature vectors. In this approach, each feature dimension is treated independently; thus, the feature space that is supported by the reconstructed vectors is vastly expanded, i.e., this expansion of the feature space can fill up many interior concaves, and is still restricted within the convex of the feature space. It is an underlying assumption of this approach that this expanded space is nevertheless broadly representative of the feature distribution for each class.

### 4.2. Computational Efficiency Analysis

As opposed to the parametric algorithms, including many deep learning methods, where often a large number of parameters needs to be optimized for constructing complex mathematical models, the training of instance-based classification methods (e.g., k-NN or SVM) is usually computationally efficient. On the other hand, with the size of training data increasing, the computational time will inevitably increase. Comparing between time-series and image data, due to the difference in the number of their feature dimensions, it has been found that the computation time is much shorter for the former.

In this analysis, the computational efficiency of I-ATR and its impact on classification performance is reported using the adopted two public image datasets previously introduced (FASHION-MNIST and CIFAR-10). The number of intermediate templates *K*, which indicates the preserved images for feature vector reconstruction, is also explored empirically, as this parameter has a direct impact on the overall recognition time of the proposed system.

**Figure 15:**
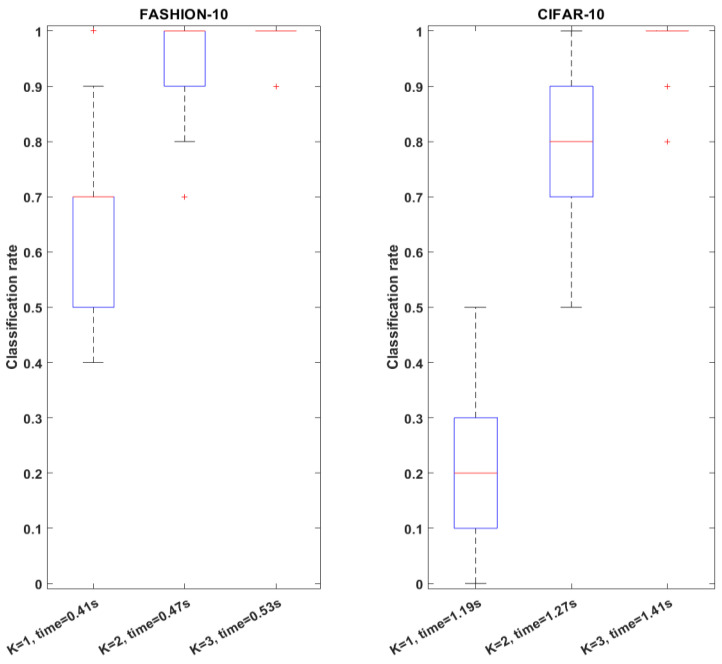
Comparative analysis of FASHION-MNIST and CIFAR-10 datasets using boxplots; each column was generated using 100 tests. The impacts of parameter *K* and recognition time on classification rate are illustrated.

Figure 15 shows the analysis of classification rate, parameter *K* and computation time. The results were obtained using an HP computer with 11th Gen Intel(R) Core i7 CPU of 2.80 GHz. As the number of preserved intermediate templates *K* for template reconstruction increases, the classification performance also increases drastically. For both the FASHION-MNIST and CIFAR-10 datasets, the mean accuracy reached more than 98% while using three images for the template reconstructing. Indeed, as the *K* increases, the time used for the classification per attempt is also increasing. For the examined 10-class classification problems, using three image samples per class for template reconstruction already reached the state-of-the-art range of performance. The computational time spent for CIFAR-10 has been found to be roughly three times longer than the FASHION-MNIST; this is due to the length of the feature vector extracted from CIFAR-10 being also three times larger.

**Figure 16:**
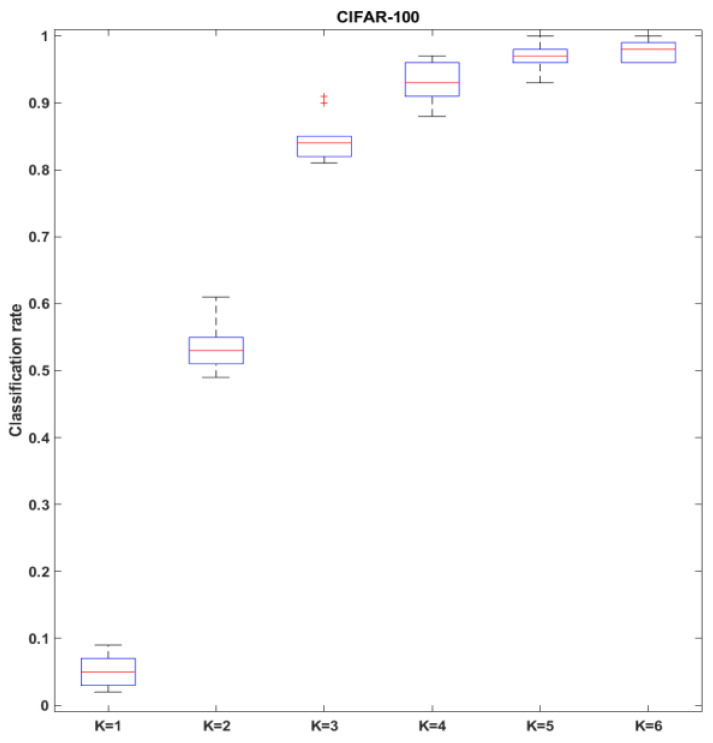
The impact of parameter *K* on classification rate for CIFAR-100 with 100 image classes. Each boxplot was generated from 100 tests.

As a further exploration, to demonstrate that the parameter *K* needs to be empirically adjusted for different datasets, the CIFAR-100 was also used to show the impact while the number of classes increased from 10 to 100. The classification rates with different *K* are illustrated in Figure 16, it indicates while the number of classes increasing, in order to maintain good performance, it may need feature elements from more image samples per class to construct the new templates to successfully distinguish the between-class differences.

The proposed approach shares certain similarities with some recent pattern recognition algorithms in its motive and conceptual principles. A number of algorithms have recently been proposed to deal with the issue of pedestrian image alignment. The alignment between the query image and the image in the database is one of the major challenges due to the variant camera angles and pedestrian postures. The pose attention transform [26] and the alignment affine transform [27] were recently developed to construct new images based on the query, which provided better matches in the database to achieve improved identification performances. Similarly, by using the reconstructed templates and query, the proposed method in this study also demonstrated much improved performance in the field of image-based pattern recognition. The proposed I-ATR method also confines the reconstruction within the “bounding box” region [27] in order to guarantee that the outcome of the feature transformations does not lose the ability to represent the original signal source. At the same time, in contrast to some approaches implemented in the image/video processing field, the proposed template reconstruction method is motivated by certain features of time-series bio-signals (such as EEG). The size of the available publicly annotated EEG data is much more limited compared to the vast quantity of image data available for machine learning. Therefore, as opposed to deep learning techniques, the I-ATR algorithm is designed to leverage a relatively small amount of data, from which to produce high-quality features in order to achieve improved recognition performance.

## 5. Conclusions

A novel instance-based template reconstruction classification algorithm (I-ATR) is presented in this work; its underlying motivation and rationale were explained and supported using a range of case studies. The proposed approach potentially generates a substantially larger volume of template candidates from the limited available data, and subsequently, a small subset with reconstructed features is retained to ensure improved class separation. The intermediate templates (Training Phase and Matching Phase) help overcome the challenge of limited data.

Extensive comparative evaluations using still images and non-stationary time-series data (EEG) were reported to demonstrate the effectiveness and robustness of the proposed method. The I-ATR classification algorithm shows superior performance in both modalities and surpassed the currently reported results for the respective databases. Specifically, the performance of the I-ATR algorithm was compared with two other popular and related instance-based classification algorithms, k-NN and SVM, using still image and EEG databases with substantially different characteristics.

The proposed method demonstrated some key advantages for image classification problems: (1) comparable performance (around 99% for CIFAR-100 dataset) is achieved with much less training time (minutes vs. hours); (2) much less data are needed compared to deep learning approaches (3 reconstructed images vs. 50,000 raw images); (3) the number of parameters that are required to be estimated is also much smaller. It was also shown to have resulted in substantial performance improvements in different evaluation scenarios. In particular, the resilience of the I-ATR to template ageing, even with input samples of limited recording length (2 to 6 min), was notable.

A few theoretical caveats could be distilled from the proposed I-ATR algorithm. Studies have indicated that the rectilinear distance metric (L_1_ norm) is consistently more preferable than distances with higher norms, such as the Euclidean distance metric (L_2_ norm) for high dimensional data mining applications [28]. Therefore, without violating the Triangle Inequality, the L_1_ norm distance measurement between elements from different instances (or feature vectors) is used in the I-ATR algorithm to construct new templates and the hyper bounding box is subsequently established in the Cartesian space. Within this hyper bounding box, each dimension from the newly constructed templates must exhibit the lowest between-class similarities possible, i.e., features distributed around the upper boundary of the hypersurface (Training Phase); as well as the shortest distances possible between the reconstructed query and template feature vectors for all the training classes, i.e., features distributed around the closest boundaries between the training and query hypersurfaces (Matching Phase). It is also worth pointing out that, although the reconstruction of the vectors in the proposed mechanism is achieved through distance measurements, other metrics, such as loss functions, may also be effective.

One focus of further work will be to explore the potential for wider applicability of the proposed approach, e.g., facial expression analysis [29], driver drowsiness prediction [30], as well as using other types of data, including those from domains other than image and time-series data, for example applications in the field of natural language processing. Also, attention will be paid to the optimization of the algorithm through the adjustment of its parameters and its sensitivity to outliers. For example, this includes further improving the efficiency of the template reconstruction mechanism, hence reducing the computational time for each classification attempt. For the scenario where a large amount of data are available, deep learning methods may be incorporated into the proposed I-ATR algorithm to further boost the performance of the approach.

## Figures and Tables

**Table 1:** Classification accuracy comparisons for the adopted two public databases. The results using the proposed method in this work are listed in the far-right column.

Datasets	Accuracy Rates for Various Techniques			
Fashion-MNIST	DARTS [12]	SAM [13]	MSDA [14]	I-ATR:
	96.91%	96.41%	96.36%	99.53%
CIFAR-10	Fractional Max-Pooling [15]	CNN [16]	LSUV [17]	I-ATR:
	96.53%	95.59%	94.16%	98.18%

**Table 2:** Longitudinal impact on EEG person identification, using the MSD.

Template Stability	Classification Accuracy (%)				
	1-NN	SVM	I-ATR Training Phase	I-ATR Matching Phase	Full I-ATR
Single Session	93.61	93.24	95.35	96.54	98.76
Multiple Sessions	11.23	10.10	53.57	59.29	85.71

## Data Availability

The data presented in this study are openly available. See [9,10,12].
